# Characterization of a novel *N*-acylhomoserine lactonase, AidP, from Antarctic *Planococcus* sp.

**DOI:** 10.1186/s12934-018-1024-6

**Published:** 2018-11-17

**Authors:** Wah Seng See-Too, Peter Convey, David A. Pearce, Kok-Gan Chan

**Affiliations:** 1 Division of Genetics and Molecular Biology, Institute of Biological Sciences, Faculty of Science, University of Malaya, 50603 Kuala Lumpur, Malaysia; 2 National Antarctic Research Centre, IPS Building, University Malaya, 50603 Kuala Lumpur, Malaysia; 3 British Antarctic Survey, NERC, High Cross, Madingley Road, Cambridge, CB3 OET UK; 4 Applied Sciences, University of Northumbria at Newcastle, Newcastle-upon-Tyne, NE1 8ST UK; 5 International Genome Centre, Jiangsu University, Zhenjiang, China

**Keywords:** Quorum quenching, Episodic positive selection, *Pectobacterium*, Soft-rot disease

## Abstract

**Background:**

*N*-acylhomoserine lactones (AHLs) are well-studied signalling molecules produced by some Gram-negative Proteobacteria for bacterial cell-to-cell communication or quorum sensing. We have previously demonstrated the degradation of AHLs by an Antarctic bacterium, *Planococcus versutus* L10.15^T^, at low temperature through the production of an AHL lactonase. In this study, we cloned the AHL lactonase gene and characterized the purified novel enzyme.

**Results:**

Rapid resolution liquid chromatography analysis indicated that purified AidP possesses high AHL-degrading activity on unsubstituted, and 3-oxo substituted homoserine lactones. Liquid chromatography–mass spectrometry analysis confirmed that AidP functions as an AHL lactonase that hydrolyzes the ester bond of the homoserine lactone ring of AHLs. Multiple sequence alignment analysis and phylogenetic analysis suggested that the *aidP* gene encodes a novel AHL lactonase enzyme. The amino acid composition analysis of *aidP* and the homologous genes suggested that it might be a cold-adapted enzyme, however, the optimum temperature is 28 °C, even though the thermal stability is low (reduced drastically above 32 °C). Branch-site analysis of several *aidP* genes of *Planococcus* sp. branch on the phylogenetic trees also showed evidence of episodic positive selection of the gene in cold environments. Furthermore, we demonstrated the effects of covalent and ionic bonding, showing that Zn^2+^ is important for activity of AidP in vivo. The pectinolytic inhibition assay confirmed that this enzyme attenuated the pathogenicity of the plant pathogen *Pectobacterium carotovorum* in Chinese cabbage.

**Conclusion:**

We demonstrated that AidP is effective in attenuating the pathogenicity of *P. carotovorum*, a plant pathogen that causes soft-rot disease. This anti-quorum sensing agent is an enzyme with low thermal stability that degrades the bacterial signalling molecules (AHLs) that are produced by many pathogens. Since the enzyme is most active below human body temperature (below 28 °C), and lose its activity drastically above 32 °C, the results of a pectinolytic inhibition assay using Chinese cabbage indicated the potential of this anti-quorum sensing agent to be safely applied in the field trials.

**Electronic supplementary material:**

The online version of this article (10.1186/s12934-018-1024-6) contains supplementary material, which is available to authorized users.

## Background

Bacteria rely on quorum sensing (QS), or bacterial cell-to-cell communication, to coordinate gene expression at high cell densities [1]. *N*-acylhomoserine lactones (AHLs) are among the best-characterized chemical signalling molecules produced by a large number of Gram-negative Proteobacteria [2, 3]. AHL-regulated QS traits are initiated when the accumulation of AHLs achieves a critical threshold concentration with sufficient molecules present to bind on the cognate transcriptional regulators, to subsequently trigger the expression of the targeted gene [4]. QS regulates the expression of up to 10% of total Open Reading Frames (ORFs) in bacterial genomes [5] and more than 20% of the proteomes [6]. Thus, QS confers a significant advantage to bacterial populations, by allowing them to behave like an organized multicellular organism [7, 8].

AHL-mediated QS has been shown to be involved in the infection phenotype of *Pseudomonas aeruginosa* [9], *Aeromonas hydrophila* [10], *Pectobacterium carotovorum* subsp. *carotovorum* [11] and a wide range of other pathogens. Some bacteria produce AHLs to regulate phenotypes that cause detrimental effects within the environment. For example, the structure of the microbial community in corals infected with Black Band disease is associated with bacteria that possess AHL-mediated QS [12, 13]. Therefore, the disruption of QS, known as quorum quenching (QQ), is of interest for its potential application in mitigating the detrimental effects caused by AHL-producing pathogenic bacteria.

There are two families of AHL-degrading QQ enzymes, AHL-lactonases and AHL-acylases. AHL-lactonases catalyse AHL ring opening by hydrolysing lactones, and examples include AiiA from *Bacillus* sp. [14], AiiB and AttM from *Agrobacterium tumefaciens* [15, 16], and QsdA from *Rhodococcus erythropolis* [17]. AHL-acylases hydrolyse the amide bond of AHLs, and reported AHL-acylases included AiiD from *Ralstonia* sp. [18], PvdQ from *P. aeruginosa* [19] and AhlM from *Streptomyces* sp. [20]. These AHL-degrading QQ enzymes have been reported in bacteria, yeast [21], and mammalian sera [22], and the genes encoding these enzymes have also been found in a metagenomic library constructed from a soil sample [23]. AHL-oxidoreductases are another family of QQ enzymes produced by *R. erythropolis* that only modify the functional group of AHLs to affect the QS activity, unlike the other AHL-degrading enzymes [17]. Various plants have also been reported to produce QS inhibitors. For instance, halogenated furanones from *Delisea pulchra* inhibit QS-mediated gene expression by promoting the degradation of the bacterial transcriptional activator [24, 25].

Unlike other antibacterial strategies that employ antibiotics and which have led to the emergence of multidrug-resistant pathogens [26], QQ does not affect bacterial viability and therefore, imposes a lower selective pressure [27]. QQ has been employed in prevention of biofilm formation on membrane bioreactors used in wastewater treatment [28], biofouling in reverse osmosis (RO) systems [29] and for the degradation of aromatic compounds [30, 31]. This strategy has also been exploited by co-culturing the heterologous system of the QQ gene or purified QQ enzyme with AHL-producing pathogens, such as *P. carotovorum*, for plant disease control [14]. Dong et al. [14] also demonstrated that heterologous expression of *aiiA* in *P. carotovorum* significantly reduced the release of AHLs and attenuated the pathogenicity of the plant pathogen.

We have previously demonstrated that the bacterial strain *Planococcus versutus* L10.15^T^, isolated from Antarctica, produces a novel class of AHL-lactonase. Homologous genes of *aidP* were identified in all type strains of *Planococcus* isolated from Antarctica, and we thus termed the gene encoding the AHL-lactonase as ‘autoinducer degrading gene from *Planococcus* sp.’ (*aidP*) [32]. In this study, we report the cloning of *aidP* from *P. versutus* L10.15^T^ and characterization of this novel AHL lactonase. We also further demonstrate that AidP is able to attenuate the virulence of *P. carotovorum* subsp. *carotovorum*, a plant pathogen that causes soft-rot disease in Chinese cabbage.

## Materials and methods

### Bacterial strains, plasmids, chemicals, and growth conditions

Bacterial strains and plasmids used in this study are listed in Additional file 1: Table S1. *Planococcus versutus* strain L10.15^T^ was isolated from a soil sample collected on Lagoon Island (Ryder Bay, Adelaide Island, maritime Antarctic), near to an elephant seal haul out and wallow area [32, 33]. *Escherichia coli* was grown at 37 °C in Luria–Bertani (LB) medium or FOC medium following the manufacturer’s instructions (Champion™ pET100 Directional TOPO^®^ Expression Kit, Invitrogen). The AHL biosensor *Chromobacterium violaceum* CV026 [34] was maintained on LB agar at 28 °C, and was used to detect short-chain (C_4_ to C_8_) HSL. Antibiotic was added at a final concentration of 100 µg/ml for ampicillin or 50 µg/ml for kanamycin, as required. *P. versutus* strain L10.15^T^ was grown in LB medium at 16 °C. The AHLs used in this study, *N*-hexanoyl-l-homoserine lactone (C_6_-HSL), *N*-heptanoyl-l-homoserine lactone (C_7_-HSL), *N*-octanoyl-l-homoserine lactone (C_8_-HSL), *N*-decanoyl-l-homoserine lactone (C_10_-HSL), *N*-docecanoyl-l-homoserine lactone (C_12_-HSL), *N*-(3-oxohexanoyl)-l-homoserine lactone (3-oxo-C_6_-HSL), *N*-(3-oxooctanoyl)-l-homoserine lactone (3-oxo-C_8_-HSL), *N*-(3-oxodecanoyl)-l-homoserine lactone (3-oxo-C_10_-HSL), and *N*-(3-oxododecanoyl)-l-homoserine lactone (3-oxo-C_12_-HSL), were obtained from Sigma Aldrich (USA) or from Prof. Paul Williams (University of Nottingham). Isopropyl-ß-d-1-thiogalactopyranoside (IPTG) was obtained from Promega (Madison, WI).

### Molecular evolution analyses, amino acid profile analysis

All gene sequences were aligned in MEGA 7 [35] using MUSCLE [36]. The multiple sequence alignment analysis was conducted using Jalview version 2 [37] and highlighted according to Clustal X (refer to Additional file 1: Table S2 for colour code interpretation). In order to investigate the presence of selective forces acting on *aidP* genes, branch-site analysis for detection of positive selection using the CodeML program within PAML 4.7 [38] was performed. The ML phylogenetic tree inferred in the analysis was constructed using sequences obtained from a BLASTn search using the *aidP* gene sequence from *P. versutus* L10.15^T^ (WP_049694637.1) (Additional file 1: Figure S1). Likelihood ratio tests (LRT) were used to test for significance between the null and alternative models (χ^2^ distribution, *p* value < 0.05). The Bayes empirical Bayes method (BEB) was used to identify positively selected sites when significant results were found [39]. The amino acid composition was calculated from the aligned region of gene sequences using CodeML analysis, and the heatmap was created using MORPHEUS (https://software.broadinstitute.org/morpheus/). The 3D structure of AidP was modelled using the automated SWISS-MODEL server [40]. Positively selected sites were highlighted using PyMOL.

### Cloning of *aidP* gene

The full length *aidP* gene from *P. versutus* L10.15^T^ was amplified by PCR using primer pair PAL200Fa (5´-CACCATGACTGGTATTATCAAGCC) and PAL200R (5′-TTATTCGTAGTATCCTTCAGTCGACT). CACC, which pairs with the overhang sequence of the pET200 vector, was added at the 5′ end of the forward PCR primer, to enable directional cloning. PCR was performed using KAPA HiFi polymerase (Kapa Biosystem, USA), with the following cycling parameters: 95 °C for 30 s, 62 °C for 30 s, and 72 °C for 1 min, for 35 cycles. The amplicons were purified using the AMPure XP-PCR purification system (Beckman Coulter, USA). The purified PCR product was cloned into the expression vector (pET-200) and transformed into *E. coli* TOP-10™. Next, the pET200-aidP was amplified in the TOP-10™ host, and extracted using the Qiagen Plasmid Mini kit (Qiagen, Germany). The pET200-aidP was then transformed into expression host *E. coli* BL21 Star™.

### Expression and purification of AidP protein

The gene was expressed in BL21 Star™ following the manufacturer’s instructions (Invitrogen, USA). Briefly, BL21 Star™ harbouring pET200-aidP were grown in LB broth at 37 °C following the manufacturer’s instructions (Invitrogen, USA). Protein expression was induced by the addition of 0.5 mM IPTG (isopropyl-ß-d-thiogalactopyranoside). The cultures were further grown in 0.5 mM IPTG (150 rpm) for 16 h. Cells were harvested by centrifugation at 5000*g* for 10 min at 4 °C. The His_6_-AidP protein was purified from the harvested cells using the Ni–NTA Fast Start Kit (Qiagen, Germany) under native conditions following the manufacturer’s instructions. Briefly, the cells were lysed using the native lysis buffer provided by the kit, filtered using Fast Start Column, and finally eluted in a final buffer of 50 mM Na phosphate, 300 mM NaCl, and 250 mM imidazole, pH 8.0. The solubility of the protein was optimized using OptiSol™ Protein Solubility Screening kit (DiLYX, USA). Next, protein in buffer was dialyzed into 1 ml of reaction buffer [500 mM Na_2_SO_4_, and 1% Tween 20 (w/v), pH6]. The protein was normalized to 1 µM and then purified by immunoprecipitation using Protein G Magnetic Beads (New England Biolabs, UK). The apparent molecular mass of the purified protein was determined by SDS-PAGE (12% [w/v]) after staining it and molecular mass standards with Coomassie brilliant blue R-250. Concentrations of the purified protein were routinely determined by the Bradford method [41], Qubit^®^ 2.0 Fluorometer (Life Technologies, USA) and Bioanalyzer 2100 (Agilent Technologies, USA) with High Sensitivity Protein Screening Chip.

### Detection of AHL-degrading activity of AidP

The mechanism of AidP AHL-degrading activity and chemical structure of products from the reaction between AidP and AHLs were determined. C_6_-HSL was first subjected to digestion by AidP, and the remaining C_6_-HSL was detected using AHL biosensor CV026, which produces purple pigment violacein in response to exogenous short chain AHLs [34]. The degradation of AHLs was further confirmed using rapid resolution liquid chromatography (RRLC, Agilent Technologies, USA). The mechanism of AidP catalysis was analysed using ultra performance liquid chromatography (UPLC) and electrospray ionization–mass spectrometry (ESI–MS). ESI–MS was performed with a 6460 triple-quadrupole instrument (Agilent Technologies, USA). Total of 1 µM of AidP was mixed with 100 µM of AHLs in 0.1 ml of reaction buffer. After 3 h incubation at 26 °C, the mixture was extracted three times with ethyl acetate, and the combined organic phase was evaporated to dryness. The RRLC analysis was performed as previously described [42] with the samples being reconstituted in 0.1 ml ACN and separated using a symmetry EC-C_18_ column (2.7 by 100 mm) (Agilent Poroshell 120 EC-C18). All experiments were conducted in triplicate, and all velocities were determined at time points by which no more than 20% of the substrates were consumed.

### Characterization of enzymatic activity of AidP

The effects of various metal ions and the metal chelating reagent EDTA on AidP activity were examined both in vitro and in vivo. For in vitro assay, 1 mM EDTA or 1 mM concentration of each metal ion (Cu^2+,^ Ca^2+^, Fe^2+^, Mn^2+^, Mg^2+^, Zn^2+^, or Co^2+^) were mixed individually with 1 µM of purified AidP protein in a reaction buffer containing 3-oxo-C_6_-HSL. After incubation at 26 °C for 3 h, the remaining 3-oxo-C_6_-HSL was measured using RRLC as described above. For in vivo assay, an overnight culture of *E. coli* harbouring the plasmid pET200-aidP was diluted into fresh LB medium with 1 mM EDTA or 1 mM concentration of each selected metal ion. 3-oxo-C_6_-HSL and IPTG were added to induce AidP over-expression after 6 h cultivation. After an additional 6 h cultivation, crude cell extracts were prepared and adjusted to the same concentration before being subjected to RRLC analysis.

### Inhibition of pectinolytic activity of *Pectobacterium carotovorum* subsp. *carotovorum* strain GS101 and PNP22

The assay was performed Chinese cabbage as described by Lojkowska et al. [43] and Uroz et al. [44]. Plant material was gently washed with sterile H_2_O, wiped with sodium hypochorite, and then dried under sterile conditions. *P. carotovorum* wild type strain GS101 was used in this assay, with *luxI* mutant *P. carotovorum* GS101, strain PNP22 grown without 3-oxo-C_6_-HSL as a negative control, or with 3-oxo-C_6_-HSL at a final concentration of 1 µM as a positive control. Briefly, strains were grown overnight at 25 °C in LB medium, suspended and diluted in PBS. The bacterial suspensions were introduced into the plant material using sterile pipette tips as (1) GS101 with or without AidP enzyme to determine the effect of AidP to attenuate the pathogenicity of the wild type *P. carotovorum*, (2) PNP22 grown with or without 3-oxo-C_6_-HSL to determine the roles of *luxI* gene in pectinolytic activity, and (3) PNP22 grown with 3-oxo-C_6_-HSL, added with AidP enzyme, to further confirms that AidP attenuate the pathogenicity via degradation of the autoinducer. The plant material was incubated at 25 °C and 90% relative humidity for 5 days. The results were assessed by direct visual inspection.

## Results

### Sequence analysis of AidP

**Fig. 1:**
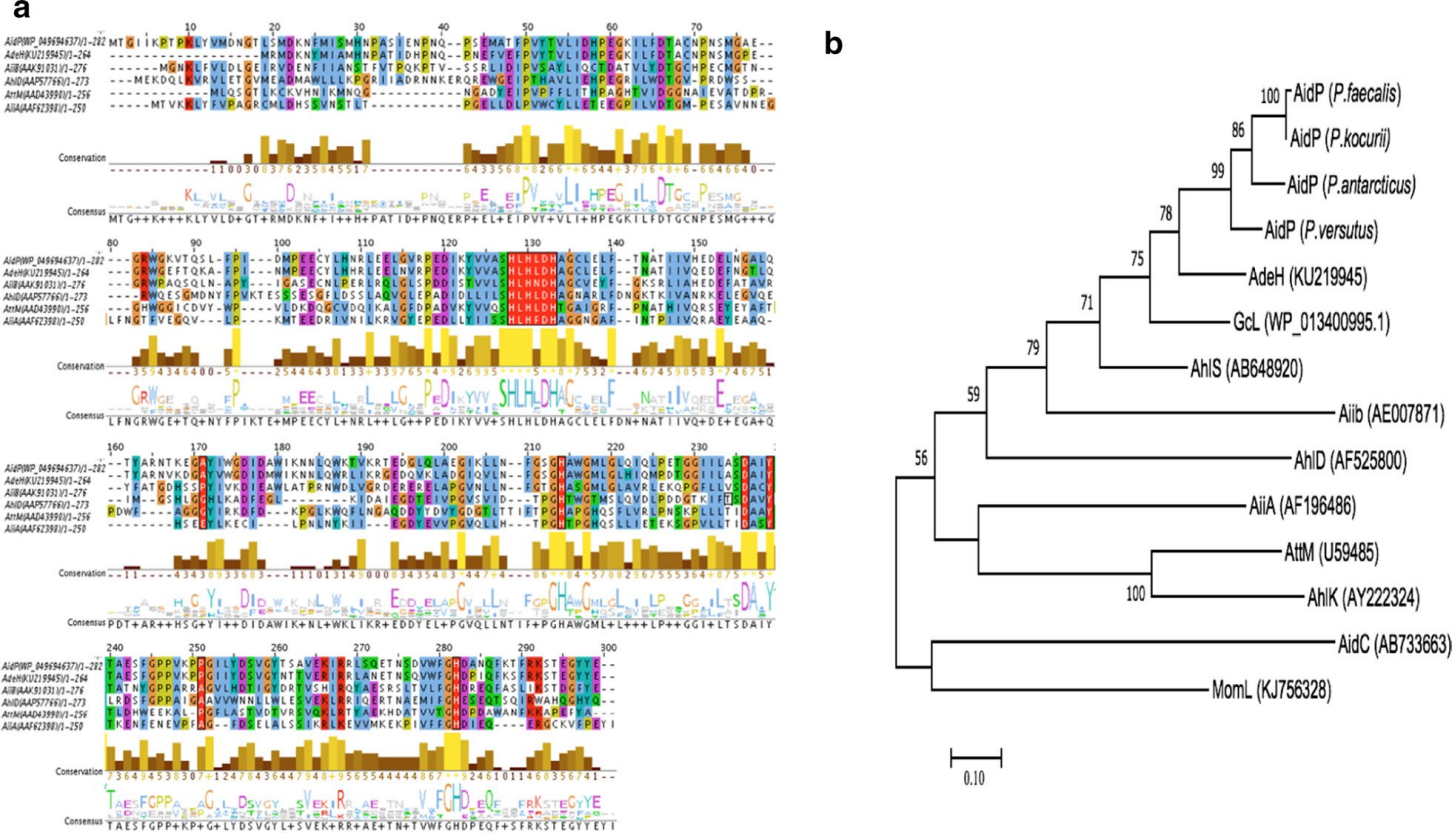
**a** Comparison of amino acid sequences from representative characterized AHL lactonases from the MBL superfamily. The amino acid sequence of AidP from *P. versutus* L10.15^T^ was compared with AdeH from *Lysinibacillus* sp. Gs50, AiiB fom *Agrobacterium fabrum* C58, AhlD from *Arthrobacter* sp. strain IBN110, AttM from *Agrobacterium tumefaciens* strain A6, and AiiA from *Bacillus* sp. 240B1. The boxed sequences are the zinc binding motif or conserved amino acid sequence involved in catalytic action. Colour code interpretation is given in Additional file 1: Table S2. **b** Phylogenetic tree of AidP from *Planococcus* sp. and other characterized AHL lactonases from the MBL superfamily

From the multiple sequence alignment analysis, we have identified the HXHXDH ~ H zinc-binding motifs, which correspond to ^117^HLHLDH^122^ ~ H^197^ in AidP, and also several functionally important amino acids based on a previous crystallographic study of AiiA-type AHL lactonase [45, 46] (Fig. 1a). These amino acids include D^121^ and D^219^, which are important in forming bridging with the ligands, H^265^ and H^122^ that interact with the zinc metal ion and the active-site residue, Y^222^, as an H^+^ donor. However, some amino acid substitutions were observed in the AidP. L^120^ and A^156^ are two amino acids that are substituted for F^107^ and E^136^ in AiiA AHL-lactonase, which form hydrogen bonds with the substrate and facilitate the interaction of the substrate with water molecule. One amino acid that is involved in the catalytic action (P^203)^, in AidC and is involved in forming the structure of the protein was substituted by E^186^ in AidP (Fig. 1a).

**Fig. 2:**
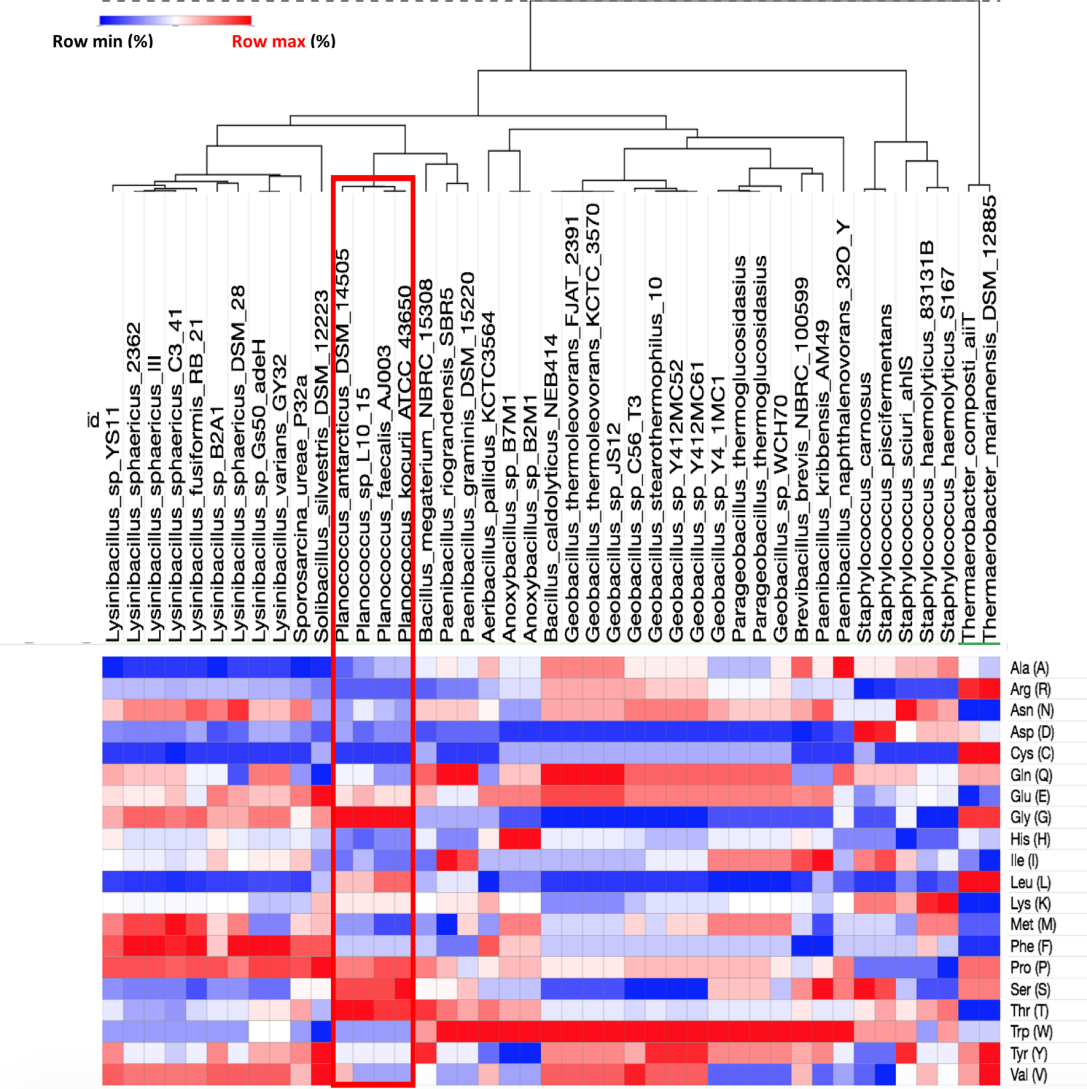
Amino acid usage analysis based on aligned region of AidP gene (box) from *Planococcus* sp. and the nearest homologous gene based on NCBI BLAST result

The phylogenetic analysis with all the known AHL lactonases from the MBL superfamily indicated that AidP is closely related to AdeH from *Lysinibacillus* and GcL from *Geobacillus* (Fig. 1b). GcL is a thermostable AHL-lactonase that is active at high temperature. However, phylogenetic analysis conducted using the NCBI BLAST search result indicated that the *aidP* gene is closely related to other hypothesized AHL lactonase gene from *Staphylococcus* spp. and *Sporosarcina ureae* (Additional file 1: Figure S1). These genes have yet to have their function characterized (in coding for AHL lactonases). Even though phylogenetic analysis on known AHL lactonases indicates that AidP has a close phylogenetic relationship with a thermophilic enzyme, amino acid substitutions at crucial sites can fundamentally change the flexibility of proteins. This also leads to some common characteristics in the amino acid composition of cold-adapted enzymes. Therefore, we conducted amino acid usage analysis on AidP and the nearest homologous genes including the thermophilic counterpart of this enzyme. The amino acid usage analysis indicated that AidP has high frequencies of Gly (G), Ser (S), Thr (T), and Leu (L) and low frequencies of Arg (A), Phe (F), and Met (M) compared to the nearest homologous genes, including the thermally-stable AHL-lactonase obtained from thermophilic bacteria such as *Geobacillus*, *Parageobacillus* and *Themaerobacter* (Fig. 2, Additional file 2: Table S3).

**Fig. 3:**
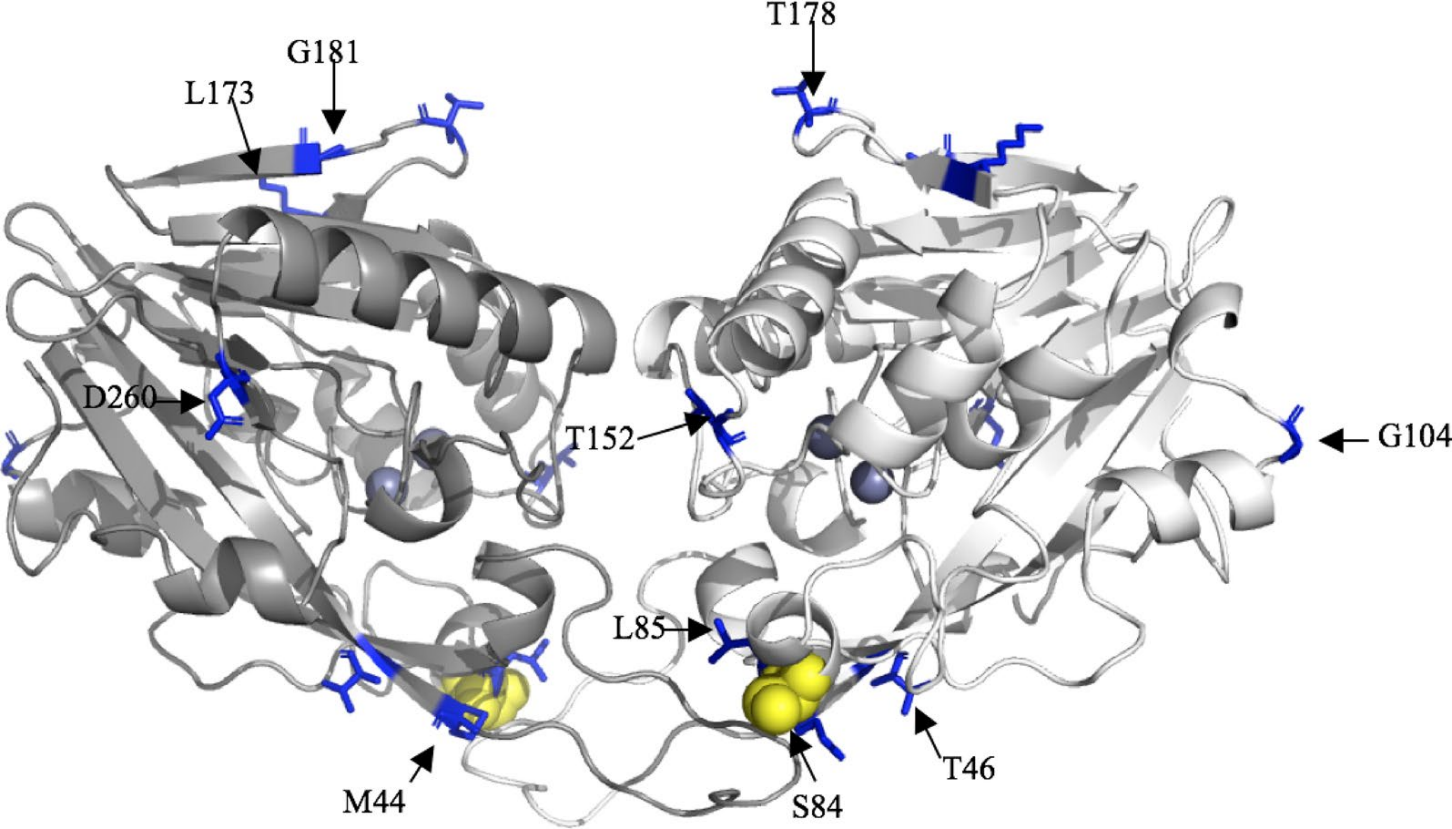
3D structure of AidP. The yellow spheres represent positively selected sites identified with Bayes Empirical Bayes (BEB) analysis at 95% and above, and the blue sticks represent positively selected sites with 50% < BEB < 95%

Since the results of phylogenetic analyses showed that AidP genes form a hitherto unknown subline in the phylogenetic trees, we hypothesized that there is selective pressure may have acted on AidP, leading to adaptation to cold environments. To find evidence of this selective pressure, we performed a branch-site analysis using CodeML, and inference was made from a phylogenetic tree constructed from the nearest gene to AidP identified in an NCBI BLAST search (Additional file 1: Figure S1). Using a branch containing four *aidP* genes from different species of *Planococcus* as foreground branch, a significant result from the likelihood ratio test (*p* < 0.05; Table 1) was obtained. A total of 11 sites under positive selection pressure were identified in Bayes Empirical Bayes analysis. One of the sites had posterior probability > 0.95, site 84S, and there were two glycine residues distant from the active site that had high posterior probability, sites 104G (0.885) and 183G (0.948) (Fig. 3). Our analysis suggested that AidP from the *Planococcus* sp. branches could be under episodic selective pressure potentially driving adaptation, since these *Planococcus* sp. strains were isolated from Antarctica [32].

**Table 1:** Summary of CodeML results for branch-site analyses for *aidP* genes and 42 homologous gene obtained from NCBI BLAST result

Model	Site class	Proportion	Background ω	Foreground ω	*lnL*	LRT Statistic	*p-value
Branch-site Null	0	0.89838	0.02528	0.02528	− 11,269.67116	4.42473	0.035422
	1	0.01171	1.000	1.000			
	2a	0.08875	0.02528	1.000			
	2b	0.00116	1.000	1.000			
Branch-site Alternative Model	0	0.91669	0.02528	0.02528	− 11,267.458798		
	1	0.01197	1.000	1.000			
	2a	0.07041	0.02528	38.61239			
	2b	0.00092	1.000	38.61239			

Foreground branch contains *aidP* genes for four *Planococcus* spp.

* *p*-value was determined using χ^2^ analysis with degree of freedom = 1

### Purification of AidP and determination of enzyme activity

**Fig. 4:**
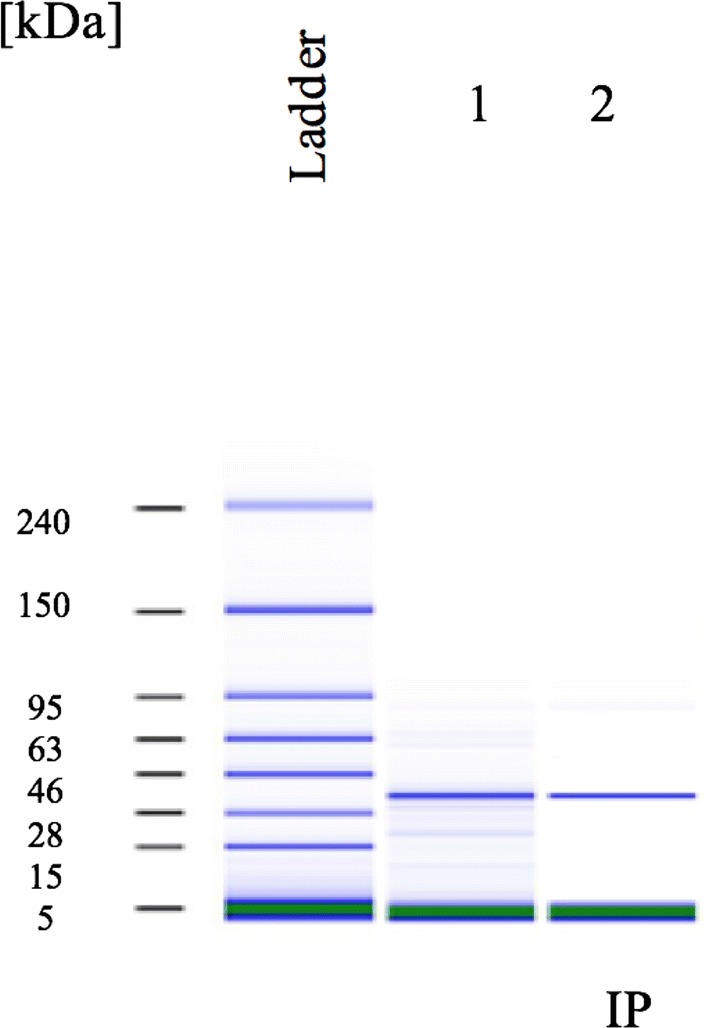
Purification of AidP protein. Lane 1, purified AidP. Lane 2, purified AidP after IP with penta-his antibody. The size of the protein was approximately 33 kDa. The amount of purified AidP was 735 ng and after IP was 702 ng

**Fig. 5:**
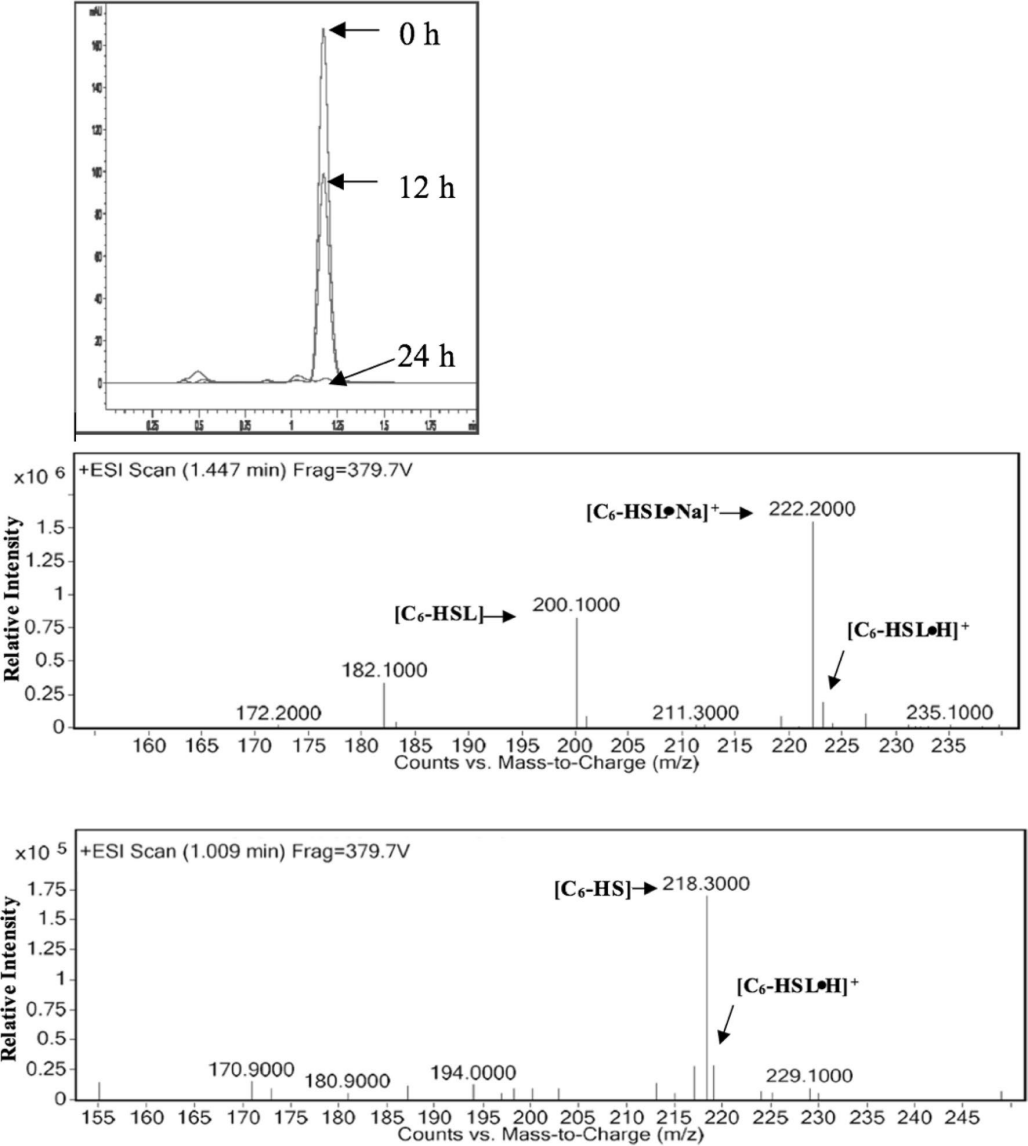
**a** RRLC of analysis digestion of C_6_-HSL by AidP (retention time of 1.1 min). **b** ESI–MS analysis of the hydrolysis product of C_6_-HSL by AidP. Retention time of 4.8 min. UPLC fragment of enzyme-digested products showed a quasimolecular (M−H) ion substrate at *m/z* of 218

The purified AidP was subjected to Bioanalyzer analysis using a high sensitivity protein assay, revealing that the protein was approximately 33 kDa in size (Fig. 4), consistent with the predicted molecular weight based on the amino acid sequence. When the AHL-degrading activity of the purified protein was examined, it completely degraded 100 µM 3OC_6_-HSL within 24 h. Results from RRLC and MS analyses indicated that AidP is an AHL-lactonase that hydrolyses the lactone ring of AHLs to acylhomoserine lactone. The RRLC profile of C_6_-HSL was characterized by a single peak with a retention time of 1.1 min. After being treated with AidP, a decrease in the milli-absorbance unit (mAU) indicated the AHL-degrading activity of AidP (Fig. 5a). ESI–MS analysis of this product revealed an (M+H) ion at an m/z of 218 (Fig. 5b), indicating the effect of AidP on C_6_-HSL, causing a mass increase of 18, corresponding to the addition of a water molecule. These results suggested that the treatment of AHL with AidP led to the cleavage of the homoserine lactone ring on the substrate, thus producing an *N*-3-oxo-hexanoyl-l-homoserine molecule.

### Substrate specificity and properties of AidP

**Fig. 6:**
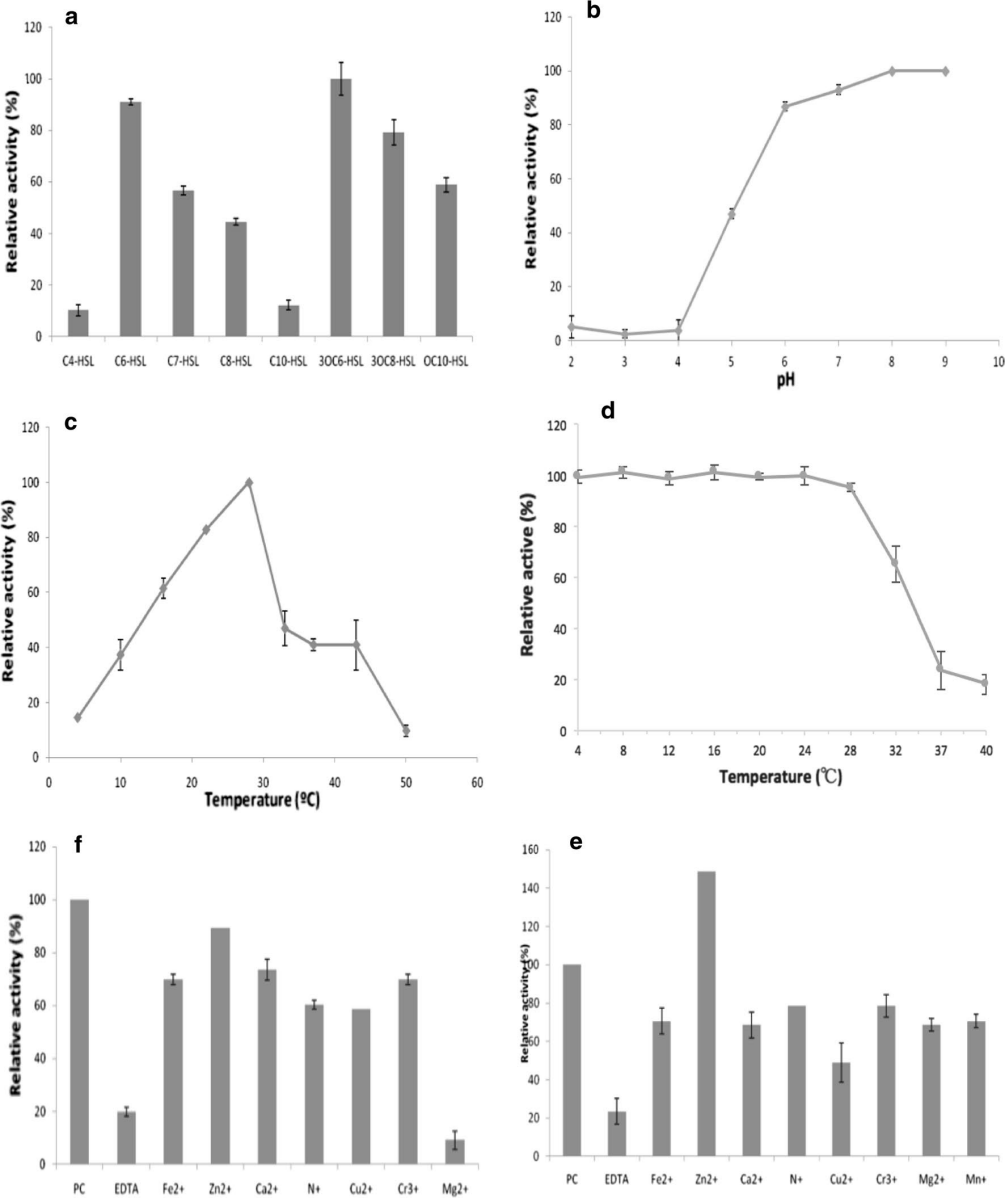
**a** Substrate specificity of purified AidP. Purified AidP was mixed with substrate solutions in buffer (pH 7). After incubation at 25 °C for 30 min, the residual AHL and its hydrolysis products were quantified by RRLC. We defined 100% relative activity as the activity toward 3OC_6_-HSL. Each incubation was replicated at least three times, and error bars indicate standard deviation. **b** Optimal pH of the AHL-degrading activity of purified AidP. Purified AidP was mixed with 3-oxo-C_6_-HSL in reaction buffers with pH ranging from pH 2 to pH 9 and incubated at 25 °C. Each incubation was replicated at least three times, and error bars indicate standard deviation. **c** Optimal temperature of AHL-degrading activity of purified AidP. Purified AidP was mixed with 3OC_6_-HSL in reaction buffer (pH 7) and incubated at temperatures ranging from 4 to 50 °C. After incubation for 30 min, the residual substrate was quantified by RRLC. We defined 100% relative activity as the activity in the reaction buffer (pH 7) at 25 °C. Control samples, which had been prepared under the same conditions but without AidP, were used to exclude the influence of autodegradation at high pH or high temperature. Each incubation was replicated at least three times, and error bars indicate standard deviation. **e** Thermal stability of AidP. Purified AidP reaction buffer (pH 7) was incubated at temperatures ranging from 4 to 40 °C for 30 min before subjected to AHLs inactivation assay with 3-oxo-C_6_-HSL. After incubation for 30 min, the residual substrate was quantified by RRLC. We defined 100% relative activity as the activity in the reaction buffer (pH 7) at 25 °C. Each incubation was replicated at least three times, and error bars indicate standard deviation. The effects of EDTA and various metal ions on the AHL-degrading activity of AidP in in vitro (**e**) and in vivo (**f**) assays. For the in vitro assay, purified AidP was incubated in reaction buffer containing substrate and a 1 mM concentration of the appropriate metal ion. After incubation at 25 °C for 30 min, the residual activity of AidP was analyzed by RRLC. For the in vivo assay, *E. coli* BL 21 Star™ harboring pET200-aidP was cultivated with 1 mM EDTA and a 1 mM concentration of the appropriate metal ion. Crude cell extracts were then prepared and used for the AHL-degradation assay. We defined 100% relative activity as the activity in the absence of EDTA or metal ions, and represented the PC (positive control). Each incubation was replicated at least three times, and error bars indicate ± 1 standard deviation

To assess the AHL substrate range of AidP, the residual AHLs present after incubation with AidP were assessed by RRLC. AidP exhibited high relative activities toward 3-oxo substituted AHLs. Nonetheless, AidP exhibited wide activities toward most AHLs tested including those with 3-hydroxy substitution, and un-substituted homoserine lactone. AidP substrate specificity was significantly affected by the length of the acyl acid chain. AidP was comparatively effective in degrading C_6_-C_10_-HSL (Fig. 6a) but showed relatively low activity towards C_4_-HSL and C_12_-HSL.

The optimal pH for AHL-degrading activity of AidP was examined using 3OC_6_-HSL as the substrate at 25 °C. AHL-degrading activity was enhanced as pH increased and reached a maximum at pH 8, while no or little activity was detected when pH was adjusted to pH 4 or lower (Fig. 6b). To determine the thermal stability of AidP, the enzyme was pre-incubated at various temperatures for 3 h and the residual enzymatic activity was then determined. The optimal temperature for the AHL-degrading activity of AidP was also examined using 3OC_6_-HSL as the substrate, and was found to be approximately 27 °C, with activity decreasing at temperatures higher than 30 °C (Fig. 6c). The relative activity of AidP at 4 °C was less than 10% of the maximum value, but it increased to 60% at 16 °C. More than 90% of the activity remained after incubation at temperatures of 30 °C or less, although activity was markedly reduced after incubation at temperatures over 32 °C (Fig. 6d).

### Zinc is essential for the AHL-degrading activity of AidP

Sequence alignment of AidP with the known AiiA-type lactonases revealed that they share the zinc-binding motifs HXHXDH ~ H (Fig. 1a). To verify whether AidP requires any cofactor for its enzymatic activity, the effects of several divalent metal ions on the AHL-degrading activity of AidP were evaluated. For in vitro assay, purified AidP was mixed with 3OC_6_-HSL in reaction buffer containing either EDTA or metal ions. When 1 mM EDTA was added to this reaction mixture, the AHL-degrading activity of AidP decreased by approximately 80% (Fig. 6d). AidP activity was also markedly inhibited by the addition of Mg^2+^. The reported metal ion inhibitor Fe^2+^ [51] showed no significant inhibitory effect on AidP. AidP activity was successfully restored to 85% of the original level by the subsequent addition of Zn^2+^, but not by any of the other metal ions tested (Fig. 6e). For in vivo assays, *E. coli* harbouring pET200-aidP was cultivated in LB medium containing EDTA or metal ions, and the AHL-degrading activity of AidP in the crude cell extracts was then determined. The AHL-degrading activity of AidP was extinguished through the addition of 1 mM EDTA but was again restored by the addition of Zn^2+^ (Fig. 6f). Furthermore, most other metal ions showed very low inhibitory effect on enzyme activity, with activity being reduced by at most 30%, with the exception of Cu^2+^, which reduced activity by more than 50%. No other metal ion stimulated AidP activity.

### AidP attenuated QS-regulated functions of plant pathogenic bacteria

**Fig. 7:**
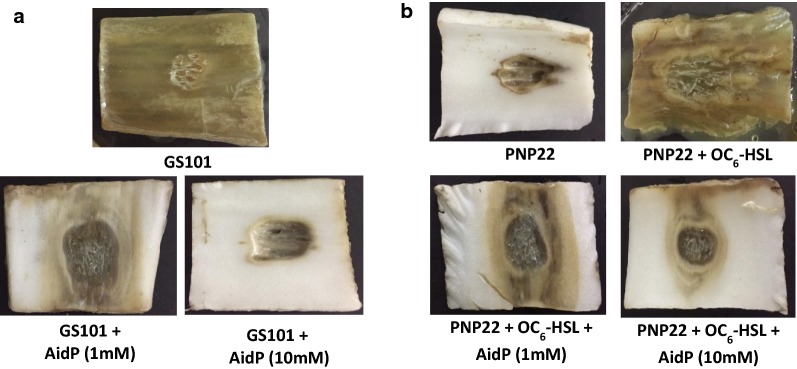
Quenching of pectinolytic activity in *P. carotovorom* strain GS101 and PNP22 by AidP. The pectinolytic inhibition assay was observed by visual inspection of maceration zones by the pathogen *P. carotovorum* strain **a** GS101 and **b** *luxI* mutant PNP22 after 5 days

The potential of AidP as an anti-QS reagent was assessed in a pectinolytic inhibition assay with Chinese cabbage. After 3 days incubation at high humidity, maceration of tissue was observed in cabbages inoculated only with GS101 (Fig. 7a). When GS101-inoculated plant material was incubated together with AidP, the area of tissue maceration was significantly reduced, and maceration was totally prevented with 10 µM AidP (Fig. 7a). The *luxI* mutant PNP22 had completely lost the pectinolytic activity (Fig. 7b), whereas the complementation of the mutant strain with exogenous AHL (3-oxo-C_6_-HSL) successfully restored pectinolytic activity (Fig. 7b). The effect of AidP in the complementation assay, was further investigated, with the data indicating that AidP had significantly reduced the pectinolytic activity of PNP22 complemented with exogenous AHL (Fig. 7b). These observations suggest that AidP is able to disrupt the QS signal from the pathogenic bacteria and hence has the potential to act as a biocontrol agent.

## Discussion

In this study, a novel AHL lactonase, AidP, was identified and characterized from the type strain of *P. versutus*. AidP is also the first reported AHL-lactonase enzyme from a bacterium isolated from Antarctica. We classified AidP as a novel AHL lactonase because of its low sequence identity compared with other AHL lactonases. Previously, we have shown that AHL-degrading *P. versutus* L10.15^T^ exhibits AHL-degrading activity at temperatures as low as 4 °C [32]. The data obtained in the current study further suggested that AidP may have structural modifications that enable its maintenance of high activity at low temperatures, thereby extending the known diversity of AiiA-type lactonases.

Amino acid composition and substitution analyses of cold-adapted enzymes have been conducted by numerous groups, to examine the structural flexibility of these enzymes at low temperature [47, 48]. A single substitution of an amino acid can stabilize/destabilize the protein structure due to the side-chain functional group of the amino acid, and drastically affect the thermal stability and thermodynamics of the protein [49, 50]. These studies have identified a number of prominent characteristics in cold-adapted enzymes, consistent with our observations on AidP, in particular having the (1) highest glycine and (2) lowest arginine composition amongst the closely related thermophilic and mesophilic homologous genes (Fig. 2). Glycine that is not in the active site (as the active site is always conserved), substantially contributes to the flexibility of the cold-adapted enzyme at low temperature [51]. Cold-adapted enzymes were observed to have low arginine composition, as arginine provides surface salt bridges or ion pairs bonding secondary structures in protein domains [52, 53]. Hawwa et al. [54] have proposed that high thermal stability in phosphotriesterase-like AHL lactonases is due to the reduced number of glycine residues in this family of AHL lactonases. *P*. *versutus* L10.15^T^ was originally isolated from Antarctic soils and we have previously demonstrated that it is capable of degrading AHLs at temperatures as low as 4 °C [32]. At low temperature, decrease in pH could affect the charge of amino acids, particularly histidine residues [55]. Thus, the apparent loss of efficiency in the isolated enzyme at 4 °C compared to the high degradation activity recorded from entire bacterial cells could indicate that the zinc-binding motifs with high histidine (HXHXDH ~ H), that are important for structural stabilization and catalysis, have been affected [56]. In the bacterial cells, we speculate there are that discrete structural modifications which allow the protein to remain active at low temperatures in the living cell (Fig. 6e, f).

Phylogenetic analysis of AidP along with other known AHL-lactonases from the MBL superfamily indicated that AidP is closely related to AdeH from *Lysinibacillus* and GcL from *Geobacillus.* AdeH has a similar active temperature range as AidP, with enzyme activity significantly reduced above 30 °C. AdeH was identified in *Lysinibacillus* isolated from soil, which is the most closely related genus to *Planococcus* that possesses AHL lactonase genes. Another closely related AHL lactonase, GcL isolated from the genus *Geobacillus*, is also closely related to *Planococcus.* However, it is a thermostable protein that is active at a high optimum temperature. These AHL lactonase genes appear to be closely related to each other with recent divergences. Variations in amino acid composition among these enzymes may explain the difference in their thermal stability. The result also prompted us to carry out branch-site analysis to test for positive selection of *aidP* genes. As the closely related genes are so diverse in terms of active temperature range, we suggest that there is selection pressure on the gene, that tunes the enzyme entropy in order to function well in cold environments. Making inference from the phylogenetic tree of these genes, we found several sites in AidP that could be under positive selection, indicating that the protein might had experienced episodic selection pressure in cold environments. Saavedra et al. [57] demonstrated how glycine substitution distant from the active-site affected the thermodynamics of a cold-adapted enzyme. This may also explains why AidP, while being closely related to a thermophilic enzyme, has distinct thermodynamic and thermal stability characteristics.

AidP exhibited the lowest optimum temperature among all the closely related AHL lactonases. Most of the AHL-lactonase genes previously identified in different taxa have optimum temperature from 20 to 50 °C, for example MomL [58], AidC [59], and AiiK [60]. AdeH, which is closely related to AidP, has a lower optimum temperature (37 °C) compared to other AHL lactonases. AidP is closely related to a number of thermally stable AHL lactonases, including GcL [61] and AiiT [62], which retains its activity at 70 °C and above.

Most known AHL lactonases, such as AiiA from *Bacillus* sp. 240B1 [63], show relatively high activity toward unsubstituted AHLs. Therefore, the high relative activities of AidP toward 3-oxo substituted AHLs compared to unsubstituted AHLs contrasts with characteristics previously reported for AHL lactonases, except AidC [59] (Fig. 6a). In contrast to MomL that has higher preference for long chain AHLs as substrate [58], AidP is similar in substrate specificity as AiiK [60], with higher activity against mid-long chain AHLs (Fig. 6a). AidP exhibited high activity toward most AHLs tested, including those with 3-hydroxy substitutions. However, in common with AiiA, AidP did not show any degrading activity against l-homoserine lactone or γ-butyrolactone.

The increase in AHL-degrading activity of AidP achieved by the addition of Zn^2+^ suggests that this ion is essential for the AHL-degrading activity of AiiA-type lactonase. The inhibition of the AHL-degrading activity of AidP by the addition of Cu^2+^ indicated that this ion is an AHL lactonase inhibitor for AidP, as has previously been reported for other AHL lactonases [63]. Other known AHL lactonases from the MBL-superfamily, including AiiA [56], AiiB [64] and MomL [58], can bind to other metal ions including Mg^2+^, which in contrast had an inhibitory effect in the in vitro assay for AidP. Nonetheless, AidP only binds to Zn^2+^ of all the metal ions tested. The effect of these metal ions on AidP is similar to those on its nearest known AHL lactonase neighbour, AdeH [65].

Most studies of AHL lactonases to date have focussed on the human pathogen, *Pseudomonas aeruginosa.* Migiyama et al. [66] has demonstrated that AHL lactonase AiiM is effective against *P. aeruginosa* in the mouse model of acute pneumonia. Tang et al. (2015) [58], on the other hand, demonstrated that MomL is effective against *P. aeruginosa* in *Caenorhabditis elegans*. AHL-lactonases such as AhlS [67, AidH 68] and AdeH [65] have also been proven effective in reducing pathogenicity of *P. carotovorum*, a species that causes soft-rot disease in various plants. When a gene encoding AHL-lactonase was transformed into *P. carotovorum*, AHL production was significantly reduced and the genes responsible for pectinolytic activity were not expressed [14, 69, 70]. A transgenic plant with the gene encoding AHL-lactonase also had substantially enhanced resistance against *P. carotovorum* [71]. These findings indicate the potential of AHL lactonases to be used as microbial antagonists.

The scientific community has however, raised concerns about the approaches used to create disease-resistant transgenic plants or crops. Firstly, resistance may be induced and developed in target bacteria even though AHL lactonases impose lower selection pressure compared to antibiotics [72]. Even though the effect of AHL lactonase is specific and potent [73], constant expression of the AHL lactonase in a plant or crop might also accelerate the emergence of resistance in the pathogen. Secondly, AHLs play crucial roles in plant defence responses, growth and development [74–76]. Therefore, expression of AHL lactonase in plant cells may also impact positive effects derived from bacteria-plant interactions. Thirdly, degradation of AHLs within plant cells itself may affect beneficial endophytes which possess AHL-mediated QS systems. Endophytes are known to improve plant rooting and nutrient uptake and enhance chlorophyll production [77]. Some endophytes produce AHLs to enhance their colonization on host plants and to counter phytopathogens [78]. Therefore, a strategy using direct application of AHL lactonases to target specific plant pathogens that produce AHLs could be recommended as treatment of infected plants, mitigating the dependency on antibiotics that could accelerate the development of antibiotic-resistant pathogens.

## Conclusions

In this study, a novel AHL lactonase from *P. versutus* was successfully cloned and expressed. It showed considerable hydrolytic activity, and we demonstrated the ability to attenuate pathogenicity of the plant pathogen *P. carotovorum* in vivo. We conclude that this anti-quorum sensing agent can be safely applied in field tests with the low thermal stability.

## Additional files

**Additional file 1: Figure S1.** Phylogenetic tree constructed using NCBI BLAST result of *aidP* gene from *P. versutus* L10.15^T^. The foreground branch is highlighted in red colour. **Table S1.** Bacterial strains and plasmids. **Table S2.** Colour coding for multiple sequences alignment analysis.

**Additional file 2: Table S3.** Amino acid usage analysis based on aligned region AidP gene and the nearest homologous gene based on NCBI BLAST result.
