# Supplementary material for: Characterization of a novel N-acylhomoserine lactonase, AidP, from Antarctic Planococcus sp
Source: Microb Cell Fact. 2018 Nov 17;17:179. doi: 10.1186/s12934-018-1024-6 (PMC6240239; doi:10.1186/s12934-018-1024-6)
Supplement: Supplementary file 1 — Additional file 1: Figure S1. Phylogenetic tree constructed using NCBI BLAST result of aidP gene from P. versutus L10.15T. The foreground branch is highlighted in red colour. Table S1. Bacterial strains and plasmids. Table S2. Colour coding for multiple sequences alignment analysis. [file 12934_2018_1024_MOESM1_ESM.docx]

**Characterization of a Novel *N*-Acylhomoserine Lactonase, AidP, from Antarctic *Planococcus* sp.**

Wah Seng See-Too^1,2^, Peter Convey^2,3^, David A. Pearce^2,3,4^, Kok-Gan Chan*^1,5^

^1^ Division of Genetics and Molecular Biology, Institute of Biological Sciences, Faculty of Science University of Malaya, 50603 Kuala Lumpur, Malaysia

^2^ National Antarctic Research Centre, IPS Building, University Malaya, 50603 Kuala Lumpur, Malaysia

^3^ British Antarctic Survey, NERC, High Cross, Madingley Road, Cambridge CB3 OET, UK

^4^Applied Sciences, University of Northumbria at Newcastle, Newcastle-upon-Tyne NE1 8ST, UK

^5^ International Genome Centre, Jiangsu University, Zhenjiang, China

*Corresponding author: Institute of Biological Sciences (Division of Genetics and Molecular Biology), Faculty of Science, University of Malaya, 50603 Malaysia. Tel: +603-79675162, Fax: +603-79674509. Email: [kokgan@um.edu.my](mailto:kokgan@um.edu.my)


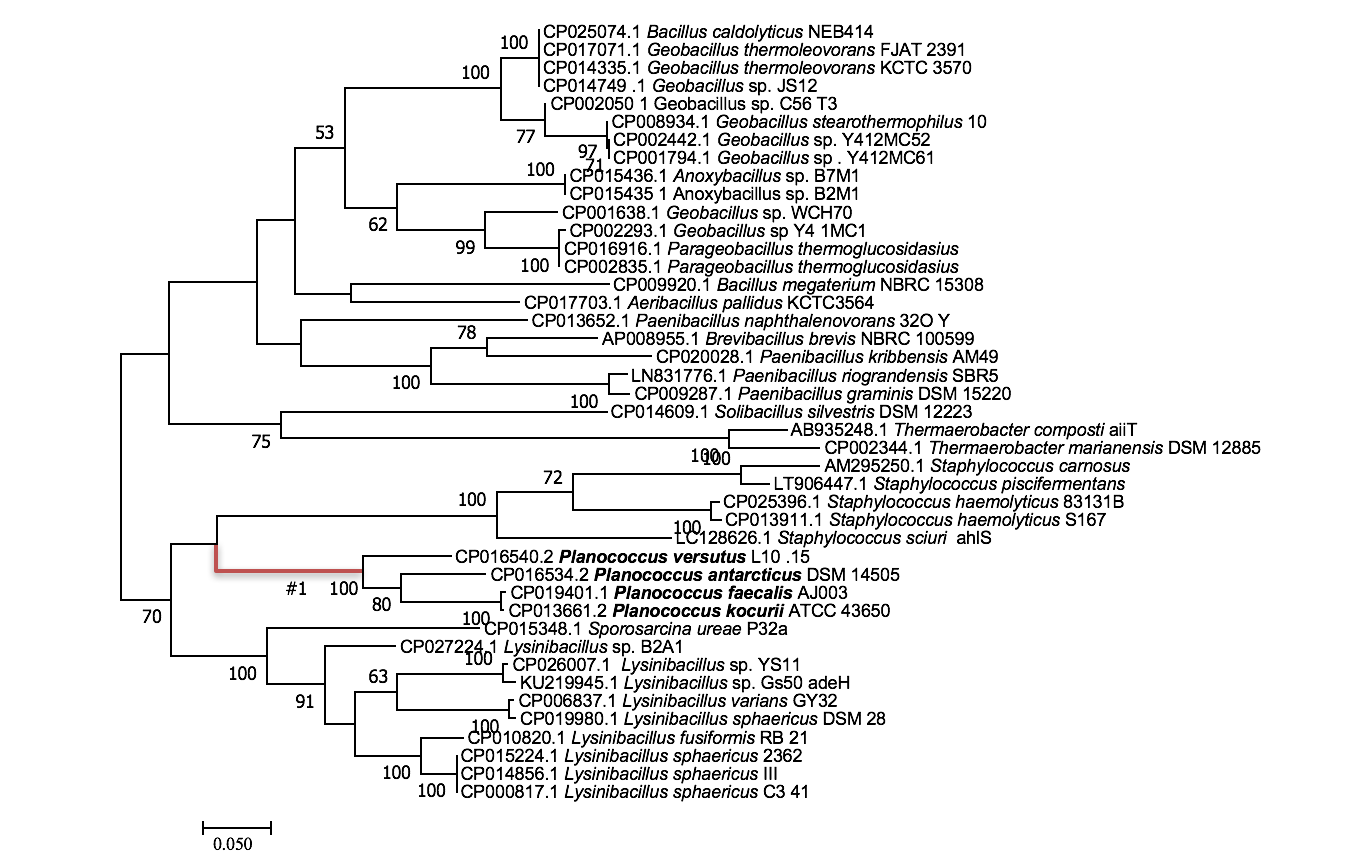


Figure S1 Phylogenetic tree constructed using NCBI BLAST result of *aidP* gene from *P. versutus* L10.15^T^. The foreground branch is highlighted in red colour.

1. **Table S1 Bacterial strains and plasmids**

| **Strain or plasmid** | **Description** | **Source** |
| --- | --- | --- |
| Strains |  |  |
| ***E.coli*** |  |  |
| Top 10 | Cloning strain | Invitrogen |
| BL21 Star^TM^ | Expression strain | Invitrogen |
| *Planococcus versutus* L10.15^T^ | AHL-degrading strain isolated from soil collected from Lagoon Island, maritime Antarctic | See-Too *et al.,* 2017 |
| ***Pectobacterium carotovarum*** |  |  |
| GS101 | Wild type strain of *P. carotovarum* | McGowan *et al*., 1995 |
| PNP22 | *LuxI* (*carI*) mutant of strain GS101 | McGowan *et al*., 1995 |
|  |  |  |
| **Plasmids** |  |  |
| pET200 | Km^r^ ; expression vector | Invitrogen |
| pET200-aidP | Km^r^ ; pET200 carrying the *aidP* gene | This study |

**Table S2 Colour coding for multiple sequences alignment analysis**


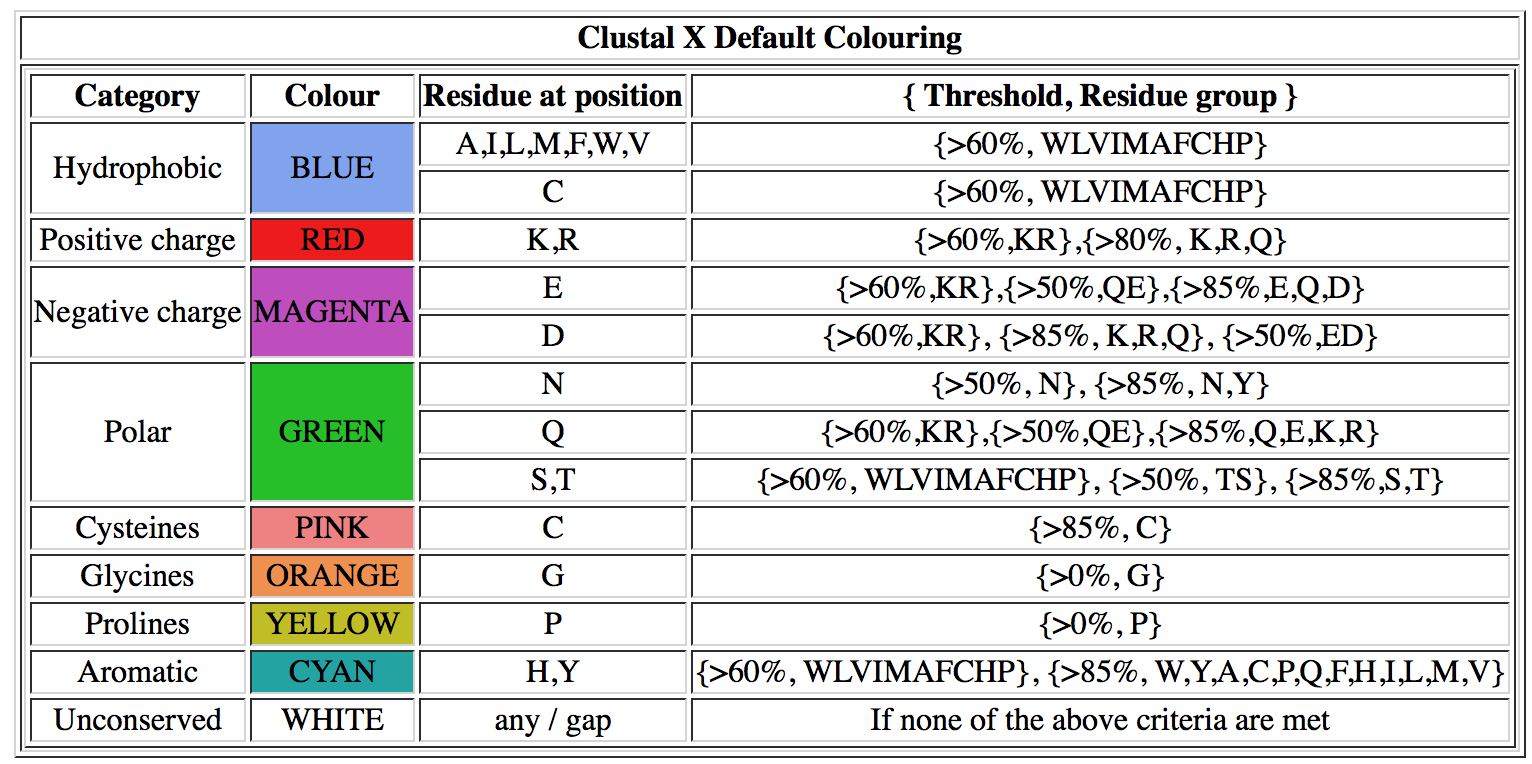


References:

1. See-Too WS, Ee R, Lim YL, Convey P, Pearce DA, Yin WF, Chan KG. *AidP*, a novel *N*-acyl homoserine lactonase gene from Antarctic *Planococcus* sp. Sci Rep. 2017;7:42968
2. McGowan S, Sebaihia M, Jones S, Yu B, Bainton N, Chan P, Bycroft B, Stewart G, Williams P, Salmond G. Carbapenem antibiotic production in Erwinia carotovora is regulated by CarR, a homologue of the LuxR transcriptional activator. Microbiology. 1995;141:541-50
